# Unveiling the New Trick for Epigenetic Drug: Radiation Enhancement of Prostate Cancer by the EZH2 Inhibitor Tazemetostat

**DOI:** 10.1111/jcmm.71330

**Published:** 2026-08-19

**Authors:** Tian‐Qi Du, Yuhong Ou, Rui‐Ji Liu, Yi Feng, Yaxun Li, Gang Chen, Xingyu Wu

**Affiliations:** ^1^ Cancer Center People's Hospital of Yangjiang Yangjiang People's Republic of China; ^2^ First School of Clinical Medicine, Health Science Center Lanzhou University Lanzhou People's Republic of China; ^3^ Department of Radiation Medicine, Biomedical Center, Institute of Modern Physics Chinese Academy of Sciences Lanzhou People's Republic of China; ^4^ Department of Oncology Guangyuan Central Hospital Guangyuan People's Republic of China; ^5^ Department of Urology, Sichuan Provincial People's Hospital, School of Medicine University of Electronic Science and Technology of China Chengdu People's Republic of China; ^6^ School of Mechanical and Energy Engineering Guangdong Ocean University Yangjiang People's Republic of China; ^7^ Department of General Surgery The First Affiliated Hospital of Jinzhou Medical University Jinzhou People's Republic of China

**Keywords:** epigenetic drug, EZH2 inhibitor, prostate cancer, radiation enhancement

## Abstract

The enhancer of zeste homologue 2 inhibitor (EZH2i) Tazemetostat (EPZ‐6438) has demonstrated antitumour efficacy in various malignancies. This study aims to evaluate its radiosensitising potential and underlying mechanisms in prostate cancer (PCa). In vitro functional assays, including wound healing, Transwell invasion, EdU proliferation, flow cytometry, and Western blotting, were employed to assess invasion, migration, proliferation, and apoptosis. DNA damage was evaluated via comet assay, immunofluorescence staining of γH2AX/53BP1 foci, and Western blot. In vivo effects on tumour progression and survival were examined in mouse models using immunohistochemistry and TUNEL apoptosis assays. Safety was also systematically evaluated. The combination of Tazemetostat and irradiation significantly suppressed PCa cell invasion, migration, and proliferation, and promoted apoptosis compared to radiation alone. In vivo, the combination group showed inhibited tumour growth, reduced invasion, induced apoptosis, and extended survival. Mechanistically, EZH2 inhibition enhanced radiation‐induced DNA damage, increased double‐strand breaks (DSBs), and impaired DSB repair capacity. The combination therapy exhibited a favourable safety profile without observable systemic toxicity. Tazemetostat effectively enhances radiosensitivity in PCa models by augmenting DNA damage and compromising repair mechanisms. These results support the clinical translation of EZH2 inhibition as a novel radiosensitising strategy for PCa patients.

## 1 Introduction

Enhancer of zeste homologue 2 (EZH2) is an evolutionarily conserved histone modifier that serves as the catalytic subunit of polycomb repressive complex 2 (PRC2). It exerts histone methyltransferase activity by catalysing trimethylation of lysine 27 on histone H3 (H3K27me3), inducing chromatin compaction and transcriptional repression of target genes [1]. PRC2, one of two highly conserved polycomb group protein core complexes, comprises three core subunits (EZH2, EED, and SUZ12) and primarily mediates gene silencing through chromatin structure remodelling [2]. Notably, emerging evidence reveals EZH2's role as a transcriptional co‐activator via non‐canonical functions involving methylation of non‐histone substrates [3]. PRC2‐independently, EZH2 methylates non‐histone targets, directly binds proteins, and activates downstream genes [4, 5, 6]. In MLL‐rearranged leukaemia, EZH2 interacts with cMyc and p300 through its transactivation domain, activating AML‐associated genes including cMyc [7]. cMyc amplification in cancers may potentiate this mechanism, enhancing EZH2's non‐canonical functions [7]. Consequently, EZH2 functions as a master regulator promoting cancer cell survival, proliferation, invasion, and therapy resistance by reprogramming critical gene expression [8, 9].

Substantial evidence implicates EZH2 in the pathogenesis of diverse malignancies, including breast, colorectal, and ovarian cancers, lymphomas, and hepatocellular carcinoma [10, 11]. In PCa, EZH2 directly occupies the androgen receptor (AR) promoter to activate transcription, independently of PRC2 and its methyltransferase activity [12]. Another study demonstrates EZH2's dual regulation of mitotic regulator CDCA8: Beyond relieving let‐7b‐mediated transcriptional repression via canonical H3K27me3 marking, EZH2 promotes E2F1‐driven CDCA8 transcription by enhancing E2F1 autoregulation [12]. This indicates synergistic cooperation between methylation‐dependent and ‐independent EZH2 functions in CDCA8 activation. Paradoxically, EZH2 also modulates translational and post‐translational processes, diverging from its repressive role, by directly methylating rRNA 2'‐O through fibrillarin binding [13]. This paradigm shift expands EZH2's functional repertoire from classical transcriptional repression to positive translational regulation. Furthermore, EZH2 stabilises DNA damage‐binding protein 2 (DDB2) by impeding its ubiquitination, thereby promoting nucleotide excision repair in cisplatin‐resistant non‐small cell lung cancer (NSCLC) via a PRC2‐independent mechanism [14]. Collectively, EZH2's oncogenic functions involve: (i) PRC2‐dependent H3K27 methylation, (ii) PRC2‐dependent non‐histone methylation, (iii) PRC2‐independent gene transactivation.

Given its pivotal oncogenic role, multiple small‐molecule EZH2 inhibitors have been developed—including DZNep, GSK126, GSK343, GSK503, CPI‐1205, CPI‐0209, SHR2554, XNW5004, mevrometostat (PF‐06821497), valemetostat (Ezharmia; EZH1/2 dual inhibitor), and tazemetostat (Tazverik; EPZ‐6438). Currently, valemetostat and tazemetostat are the only clinically approved EZH2 inhibitors (EZH2i). While valemetostat is indicated for relapsed/refractory adult T‐cell leukaemia/lymphoma (R/R ATL), tazemetostat is approved for epithelioid sarcoma (ES) and relapsed/refractory follicular lymphoma (FL). Nevertheless, clinical investigations of EZH2i for other haematologic and solid tumours are rapidly expanding. For PCa, according to incomplete statistics, active clinical trials include NCT06568094, NCT06551324, NCT06022757, NCT04846478, NCT04388852, NCT04179864, NCT04104776, and NCT03460977.

Radiation induces diverse DNA lesions through direct and/or indirect mechanisms, encompassing base damage, single‐strand breaks (SSBs), double‐strand breaks (DSBs), and DNA interstrand crosslinks. The reparability of these lesions determines cellular fate. Previous studies demonstrate that a 1‐Gy dose generates approximately 10,000 base lesions, 1000 SSBs, and 40 DSBs per cell. Although DSBs constitute a minor fraction, they inflict the most severe damage, as a single unrepaired DSB can trigger permanent growth arrest or cell death [15]. Consequently, a cell's capacity to recognise and respond to DNA damage fundamentally governs its radiosensitivity or radioresistance [16, 17].

EZH2 is frequently overexpressed in malignancies, and its association with tumour radiosensitivity has been explored. Emerging evidence indicates that EZH2 suppression enhances radiosensitivity in bladder cancer by impairing DNA damage repair, reducing stemness, and disrupting autophagic flux [18]. Similarly, miR‐126‐5p‐mediated EZH2 downregulation elevates KLF2 expression while suppressing BIRC5, thereby inhibiting proliferation and migration and augmenting radiosensitivity in lung adenocarcinoma [19]. Critically, EZH2 inhibition has been shown to radiosensitise PCa cells in vitro [20]. However, to the best of our knowledge, no study has comprehensively evaluated whether EZH2i can radiosensitise PCa in both in vitro and in vivo settings.

## 2 Materials and Methods

### 2.1 Cell Lines and Cell Culture

RM‐1 and PC‐3 cells were grown in Roswell Park Memorial Institute (RPMI) 1640 supplemented with 10% fetal bovine serum (FBS). All cultures were maintained at 37°C under 5% CO_2_.

### 2.2 Cell Irradiation

Irradiation was delivered using an X‐ray irradiator system (X‐Rad 225XL, Precision X‐ray, Madison, Connecticut, USA) at 225.0 kVp peak voltage/13.3 mA tube current (actual dose rate: 281–288 cGy/min; F1 2 mm Al filter; SSD = 40 cm). To minimise environmental interference, all experimental samples were transferred simultaneously into the irradiation chamber. Prior to irradiation, samples were labelled and sealed with parafilm. All irradiation was conducted at room temperature (RT).

### 2.3 CCK‐8 Assay

Single‐cell suspensions were seeded in 96‐well plates at 4000 cells/well (200 μL/well) with six replicates per group. After interventions (for EZH2i + irradiation groups: Pretreated with EZH2i 2 h prior to irradiation—this protocol applies throughout subsequent experiments unless otherwise specified), plates were returned to the incubator. At 48 h post‐treatment, 10 μL CCK‐8 reagent was added to each well, followed by 2 h incubation. Absorbance was measured at 450 nm.

Cell viability and inhibition rate were calculated as:

(1) $$\text{Viability}\;\left(\%\right)=\left[\left(\mathrm{As}-\mathrm{Ab}\right)/\left(\mathrm{Ac}-\mathrm{Ab}\right)\right]\times 100\%$$

(2) $$\text{Inhibition rate}\;\left(\%\right)=\left[\left(\mathrm{Ac}-\mathrm{As}\right)/\left(\mathrm{Ac}-\mathrm{Ab}\right)\right]\times 100\%$$

where *As* represents the OD value of the test well (experimental group), *Ab* represents the OD value of the blank well (containing culture medium and CCK‐8), *Ac* represents the OD value of the control well (containing cells, culture medium, and CCK‐8).

### 2.4 Clone Formation Assay and Survival Fraction

The cells were incubated in 6‐well plates and maintained in RPMI 1640. Following treatment with varying radiation doses and drug concentrations, cells were continuously cultured. After 48 h, the medium was replaced with drug‐free medium. On day 10, cultures were terminated. Supernatants were discarded, and cells were rinsed twice with PBS. Subsequently, 1 mL of absolute methanol was added per well to fix cells at RT for 20 min, followed by removal. Cells were then stained with 1 mL of 0.1% crystal violet solution for 20 min at RT. After discarding the dye, wells were rinsed until background clearance was achieved and air‐dried at RT. Colony numbers per group were quantified.

Survival fraction (SF) curves were fitted using the linear‐quadratic (L‐Q) model:

(3) $$\mathrm{SF}=e^{-\left(\mathrm{\alpha D}+\mathrm{\beta D}\wedge 2\right)}$$

where *D* represents radiation dose (Gy), and *α* (Gy^−1^) and *β* (Gy^−2^) represent radiobiological sensitivity parameters.

### 2.5 Sensitiser Enhancement Ratio and Dose Reduction Rate

The sensitiser enhancement ratio (SER) was calculated as:

(4) $${\mathrm{SER}}_{10\%}={\text{D}}_{10}^{\mathrm{DMSO}}/{\text{D}}_{10}^{\mathrm{DEZH2i}}$$

where *D*_*10*_ represents the radiation dose required to achieve 10% SF (SF_10%_). Subsequent references to SER specifically indicate SER_10%_.

The dose reduction rate (DRR) was calculated as:

(5) $$\mathrm{DRR}=\left[1-\left({\text{D}}_{10}^{\mathrm{DEZH2i}}/{\text{D}}_{10}^{\mathrm{DMSO}}\right)\right]\times 100\%$$

### 2.6 Radiation‐Drug Synergistic Effect

A dose‐effect matrix was constructed using cell inhibition rates calculated via CCK‐8 assay (Equation 2). Synergy scores (δ) were subsequently quantified and visualised using SynergyFinder. The radiation dose‐drug concentration combination corresponding to the peak δ score region was identified as the optimal synergistic regimen.

### 2.7 Wound Healing Assay

5 × 10^5^ cells were seeded per well in 6‐well plates. Upon reaching 90% confluency, monolayer scratches were created perpendicular to the plate surface using a 200 μL pipette tip guided by a straight edge. Culture medium was aspirated, followed by three gentle washes with sterile PBS. Wells were then replenished with either drug‐containing medium or serum‐free (FBS‐deprived) medium according to experimental groups. Wound healing was imaged at 0, 12, and 24 h post‐treatment.

### 2.8 Transwell Invasion Assay

Matrigel was thawed overnight at 4°C with pre‐cooled consumables (tips/EP tubes/serum‐free medium, SFM) transferred to ice pre‐experiment. After a 1:8 dilution in chilled SFM, 50 μL Matrigel was vertically loaded into Transwell inserts and polymerised (30 min, 37°C). Inserts were hydrated with 50 μL 0.1% BSA/SFM (30 min, 37°C). Log‐phase cells were harvested (trypsinisation/centrifugation), washed twice with PBS, and resuspended in 0.1% BSA/SFM at 1.5 × 10^5^ cells/mL. Cell suspension (200 μL) was seeded into inserts with 600 μL of 15% FBS medium in lower chambers. Following treatments (drugs/radiation), cells were cultured for 24 h. Inserts were PBS‐washed twice, non‐migrated cells removed with a cotton swab, fixed in methanol (20 min), stained with 0.1% crystal violet (20 min), rinsed until clear, air‐dried, and imaged for quantification.

### 2.9 EdU Cell Proliferation Assay

Log‐phase cells were seeded uniformly in 24‐well plates. Following drug/radiation treatments, plates were cultured for 48 h. Cells were incubated with 500 μL EdU working solution (500:1, medium: EdU) for 2 h, fixed with 4% paraformaldehyde (30 min, RT), and permeabilised with 0.5% Triton X‐100 (10 min). Apollo staining (500 μL, 30 min, dark) preceded three 10‐min Triton X‐100 washes and two 5‐min methanol rinses. After Hoechst 33,342 staining (30 min, dark) and PBS washes, antifade mountant was applied for fluorescence microscopy imaging and Fiji quantification.

### 2.10 Flow Cytometry

Log‐phase cells were seeded uniformly in 6‐well plates. Following 48 h of culture, media was collected, and cells were PBS‐washed (4°C, ×2). After digestion with 500 μL EDTA‐free trypsin (terminated using collected media), cell suspensions were centrifuged (1,200 rpm, 3 min). Pellets were PBS‐washed twice, resuspended in 200 μL 1× binding buffer, and aliquoted into labelled 1.5 mL tubes. Experimental samples received either 10 μL Annexin V‐FITC (single‐stain control) or 5 μL PI (single‐stain), while all other samples were dual‐stained with both reagents. After 15 min dark incubation (RT), samples were gently resuspended and analysed via flow cytometry (488 nm excitation).

### 2.11 Immunofluorescence Staining

Log‐phase cells were seeded on glass‐bottom dishes and cultured overnight. Post‐treatment (drug/radiation), samples were fixed in 4% PFA (30 min, ice), permeabilised with 0.5% Triton X‐100 (15 min, ice), and blocked with 10% goat serum (1 h, RT). Primary antibodies (v/v; γH2AX, 1:200; 53BP1, 1:200) were applied overnight (4°C) followed by fluorescent secondary antibody (v/v; 1:200, 90 min, dark). After DAPI counterstaining, samples were imaged by a confocal microscopy system (LSM‐700, Carl Zeiss Microscopy GmbH, Jena, Germany). All washes used PBS/TBST (3 × 10 min) between steps.

### 2.12 Comet Assay

1% normal melting agarose (NMA) and 0.7% low melting agarose (LMA) were prepared in PBS (95°C/75°C dissolution, respectively), equilibrated at 45°C/37°C. Harvested cells (trypsinised + supernatant) were PBS‐washed and resuspended at 1 × 10^6^ cells/mL. Pre‐warmed slides received 30 μL NMA (45°C), solidified (10 min, 4°C). Cell suspension (10 μL, 10^4^ cells) mixed with 75 μL LMA (37°C) was layered (70 μL) over NMA, then solidified (10 min, 4°C). Slides were lysed (9:1 lysis buffer: DMSO, 2 h, 4°C), alkaline‐unwound (40 min, RT), electrophoresed (25 V, 25 min, 0°C–4°C), neutralised (3 × 5 min, 4°C), stained with PI (20 μL, 15 min, dark), and imaged. Tail DNA%, Olive and Extent Tail Moments were quantified via OpenComet (Fiji).

(6) $$\text{Tail}\;\mathrm{DNA}\%=\left(\text{Tail}\;\mathrm{DNA}\;\text{Intensity}/\text{Cell}\;\mathrm{DNA}\;\text{Intensity}\right)\times 100\%$$

(7) Olive Tail Moment=TailDNA%×Tail Moment Length

(8) Extent Tail Moment=TailDNA%×Length of Tail

### 2.13 Western Blot Assay

Cells were lysed in RIPA/protease inhibitor (30 min, ice), centrifuged (12,000 rpm, 10 min, 4°C), and supernatants stored at −80°C. Protein concentrations were determined by BCA assay (562 nm), normalised, mixed 4:1 with 5× loading buffer, and denatured (10 min, 100°C). Proteins were electrophoresed through SDS‐PAGE gels (80–120 V), transferred to methanol‐activated PVDF (110 V, 70 min, 0°C–4°C), blocked with 5% skim milk (1 h, RT), and incubated with primary antibodies (v/v; β‐Actin, 1:2000; EZH2, 1:5000; H3, 1:2000; H3K27me3, 1:500; MMP‐2, 1:1000; MMP‐9, 1:1000; E‐cad, 1:10,000; Bcl‐2, 1:5000; Bax, 1:5000; cCasp3, 1:500; γH2AX, 1:1000; 53BP1, 1:1000; 4°C, overnight) and HRP‐secondaries (1 h, RT) with TBST washes. Signals were detected by ECL and quantified (Fiji) against β‐actin.

### 2.14 Mouse Models

Male 6‐week‐old mice were maintained under specific pathogen‐free (SPF) conditions at 24°C ± 2°C with 50%–70% humidity and a 12 h/12 h light–dark cycle. Mice were housed five per cage with ad libitum access to autoclaved feed, bedding, and water. After one week of acclimatisation, the right dorsal flank region was selected as the inoculation site. The skin was disinfected with 75% ethanol swabs, and 100 μL of a homogeneously resuspended cell suspension (1 × 10^7^ cells/mouse) was subcutaneously injected using a sterile 1 mL syringe via an “L”‐shaped needle insertion technique. RM‐1 cells were inoculated into C57BL/6J mice, while PC‐3 cells were inoculated into BALB/c‐nu mice. The animal study protocol was approved by the Animal Research and Ethics Committee of the Biomedical Center, Institute of Modern Physics, Chinese Academy of Sciences (No. 2023 (023)).

### 2.15 Animal Grouping and Intervention

Both BALB/c‐nu and C57BL/6J mouse models were allocated into four experimental groups (*n* = 5/group) following randomisation when tumour volume (TV) reached 50 mm^3^: (1) Control group (vehicle treatment), (2) EZH2i group (EPZ‐6438 monotreatment), (3) IR group (irradiation monotreatment), and (4) EZH2i + IR combination group. Localised tumour irradiation (4 Gy/fraction) was administered on days 0, 3, and 6 using a calibrated X‐ray irradiator. EZH2i (EPZ‐6438, 50 mg/kg) was delivered via daily intraperitoneal injection commencing on day 0. All interventions continued until experimental endpoints (Figure S1A).

### 2.16 Animal Irradiation

When TV reached approximately 100 mm^3^, abdominal skin was sterilised with 75% ethanol swabs followed by intraperitoneal injection of 1% pentobarbital sodium (50 mg/kg) using a sterile 1 mL syringe for anaesthesia. Irradiation was delivered using X‐Rad 225XL Irradiator System at 225.0 kVp peak voltage/13.3 mA tube current (actual dose rate: 192–198 cGy/min; F3 0.3 mm Cu filter; SSD = 40 cm). Non‐tumour body regions were shielded with lead blocks while limbs and tails were immobilised with medical tape prior to targeted tumour irradiation.

### 2.17 Tumour Measurement

Commencing post‐inoculation, tumour maximal diameter (length, *L*) and minimal diameter (width, *W*) were measured every 2 days using electronic callipers, with body weight recorded via electronic balance. TV was calculated as:

(9) $$\mathrm{TV}\;\left({\mathrm{mm}}^{3}\right)=\left(\pi \cdot L\cdot W^{2}\right)/6$$

Relative tumour volume (RTV) was calculated as:

(10) $$\mathrm{RTV}=\mathrm{TV}/{\mathrm{TV}}_{0}$$

where *TV*_*0*_ denotes baseline volume at day 0 (D_0_). Tumour growth inhibition (TGI) was calculated as follows:

(11) $$\mathrm{TGI}=\left[\left(1-{\mathrm{RTV}}_{\text{treatment}}\right)/{\mathrm{RTV}}_{\text{control}}\right]\times 100\%$$

Humane endpoints requiring euthanasia by cervical dislocation (recorded as death in survival analysis) were triggered if any criterion was met:

1. Projected tumour volume > 2,000 mm^3^ at next measurement
2. Projected maximal diameter > 20 mm at next measurement
3. Ulceration/necrosis at tumour site
4. Significant mobility impairment

Upon euthanasia, tumours were carefully dissected intact, with volume re‐measured and weight recorded.

### 2.18 Immunocytochemistry Staining

Tissue sections were baked (1 h, 60°C), deparaffinised in xylene twice, and hydrated through graded ethanol (100–75%) to distilled water. Antigen retrieval was performed in boiling citrate buffer followed by cooling and PBS washes. Endogenous peroxidase blocking preceded permeabilisation with 0.1% Triton X‐100. After blocking with goat serum (30 min, RT), primary antibodies were applied for 2 h at RT: Ki67 (Mouse 1:2000; Human 1:3000), Bax (1:2000), Bcl‐2 (Mouse 1:500; Human 1:200), cCasp3 (1:50), MMP‐2 (1:300), MMP‐9 (1:300), E‐cadherin (1:5000). Biotinylated IgG and HRP‐streptavidin were sequentially incubated (15 min each). DAB development, haematoxylin counterstaining with bluing, ethanol dehydration (80–100%), xylene clearing, and resin mounting preceded microscopic imaging.

### 2.19 TUNEL Staining

Deparaffinised tumour sections were encircled with a hydrophobic barrier, treated with Proteinase K (20 min, 37°C) followed by permeabilisation buffer (20 min, RT), with PBS washes (5 min × 3) after each step. TUNEL reaction mix (TdT:dUTP = 1:9) was applied and incubated in a humidified chamber (2 h, 37°C). After PBS washes (5 min × 3), DAPI counterstaining proceeded (10 min, RT, dark). Following final PBS washes (5 min × 3), sections were mounted with antifade reagent for fluorescence microscopy imaging.

### 2.20 H&E, Masson, and Sirius Red Staining

#### 2.20.1 H&E Staining

Deparaffinised sections were stained with haematoxylin (5 min), differentiated in 1% HCl/ethanol (30 s), counterstained with eosin (3 min), then dehydrated through graded ethanols (80%, 95%, 100%; 5 min each) and cleared in xylene prior to resin mounting for light microscopy.

#### 2.20.2 Masson Staining

Sections underwent sequential staining: Weigert's iron haematoxylin (5 min), Ponceau S acid fuchsin (5 min), acetic acid treatment (5 s), and aniline blue (5 min), with 0.2% acetic acid rinses; followed by ethanolic dehydration (95% I/II, 100% I/II; 5 min each) and xylene clearing before resin mounting.

#### 2.20.3 Sirius Red Staining

After Weigert's iron haematoxylin staining (10 min) and Picro‐Sirius red incubation (30 min), sections were rapidly dehydrated (75%, 95%, 100% ethanol; 1 min each), cleared in xylene, and mounted for polarised light imaging.

### 2.21 Peripheral Blood Tests

On day 14, submandibular vein puncture was performed after disinfection with 75% ethanol swabs. A total of 180 μL of venous blood was collected into EDTA tubes: 80 μL for three‐part differential complete blood count (CBC) and 100 μL for biochemical analysis. Tubes were gently inverted 10 times to ensure anticoagulant mixing. CBC samples were immediately analysed, while biochemical samples underwent centrifugation (3,000 rpm, 15 min, 4°C) to obtain plasma supernatant. Key biochemical markers included: Alanine aminotransferase (ALT), Aspartate aminotransferase (AST), Blood urea nitrogen (BUN), Creatinine (Cre), Lactate dehydrogenase (LDH), and Creatine kinase (CK). Assays were performed using commercially available kits per manufacturer protocols.

### 2.22 Statistical Analysis

All continuous variables were derived from 3 to 5 independent biological replicates, with error bars denoting mean ± standard deviation (SD), unless otherwise specified. Normality and homoscedasticity were confirmed via Shapiro–Wilk and Brown‐Forsythe tests. Two‐group comparisons utilised two‐tailed unpaired Student's *t*‐test (normal distribution) or Mann–Whitney *U*‐test (non‐normal distribution). For multi‐group comparisons, one−/two‐way ANOVA with Tukey's post hoc test was applied for normally distributed data. For non‐normally distributed data, pairwise comparisons were performed using either the Bonferroni‐corrected Dunnett's *t*‐test for smaller sample sizes or the two‐stage linear step‐up procedure of Benjamini, Krieger and Yekutieli for larger sample sizes, depending on the sample size of each comparison. Survival differences were analysed by Kaplan–Meier with log‐rank test and Bonferroni correction. The significance level was preset at *p* < 0.05, with ns for not significant, * for *p* < 0.05, ** for *p* < 0.01, *** for *p* < 0.001, **** for *p* < 0.0001; all other symbols were specified in figure legends.

Details of key reagents and resources are provided in Table S1. The flowchart was shown in Figure S1B.

## 3 Results

### 3.1 In Vitro Cytotoxicity Titration

**FIGURE 1:**
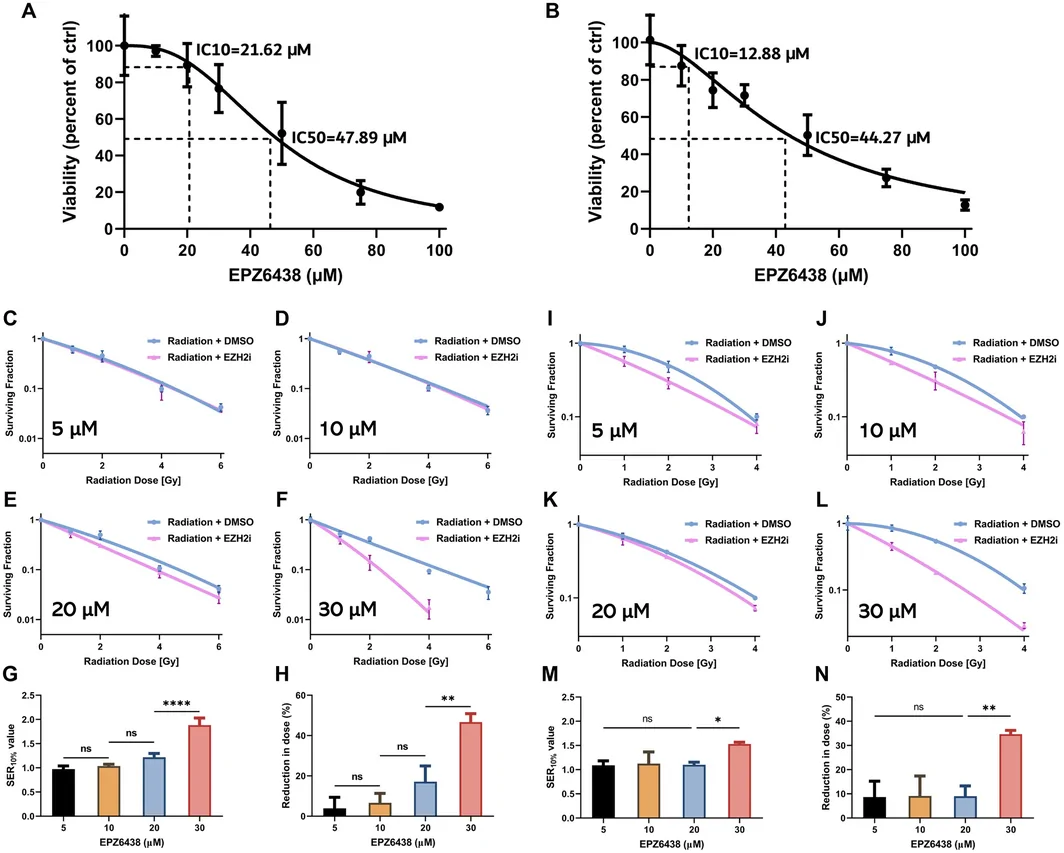
Dose–response curves of EPZ6438 in (A) RM‐1 and (B) PC‐3. Radiosensitisation to (C–H) RM‐1 and (I–N) PC‐3 by EPZ6438. (C–F, I–L) Survival curves at 5, 10, 20, 30 μM; (G, M) Sensitiser enhancement ratio; (H, N) Dose reduction rate. Ns represents no significant difference; * represents *p* < 0.05; ** represents *p* < 0.01; **** represents *p* < 0.0001.

Using CCK‐8 assays, we evaluated the cytotoxicity of EZH2i EPZ‐6438 in RM‐1 and PC‐3 cell lines. Dose‐ranging studies employed seven concentrations (0, 10, 20, 30, 50, 75, 100 μM), with cytotoxicity dose–response curves fitted to determine inhibitory concentrations. In RM‐1 cells, the 10% inhibitory concentration (IC_10_) was 21.62 μM, and the half‐maximal inhibitory concentration (IC_50_) was 47.89 μM (Figure 1A). For PC‐3 cells, corresponding values were IC_10_ = 12.88 μM and IC_50_ = 44.27 μM (Figure 1B).

### 3.2 Drug Concentration Screening

Based on IC_50_ values from Section 3.1, RM‐1 cells underwent clonogenic assays at four EPZ‐6438 concentrations (5, 10, 20, 30 μM) with radiation doses (0, 1, 2, 4, 6 Gy). Survival curves revealed minimal radiosensitisation at 5–10 μM (Figure 1C,D), while enhanced effects emerged at 20–30 μM (Figure 1E,F). Triplicate experiments quantified SER, demonstrating non‐significant SER increases from 5 to 20 μM but a significant elevation at 30 μM vs. 20 μM (Figure 1G). DRRs mirrored this trend (Figure 1H). Parallel PC‐3 assays showed negligible radiosensitisation at 5–20 μM (Figure 1I–K,M,N), with significant SER and DRR improvements only at 30 μM vs. 20 μM (Figure 1L–N).

### 3.3 EPZ‐6438 Mediated Suppression of EZH2 Activity

To validate the functional inhibition of EZH2 by 30 μM EPZ‐6438, we assessed protein expression of EZH2, H3K27me3, and total histone H3 in RM‐1 and PC‐3 cells. As shown in Figure S2, EPZ‐6438 (30 μM) significantly reduced H3K27me3 levels compared to the DMSO control, while exhibiting no effect on EZH2 or total H3 expression. This aligns with the epigenetic mechanism of EPZ‐6438, which suppresses EZH2 function by specifically attenuating H3K27 trimethylation.

### 3.4 Radiation Dose Screening

**FIGURE 2:**
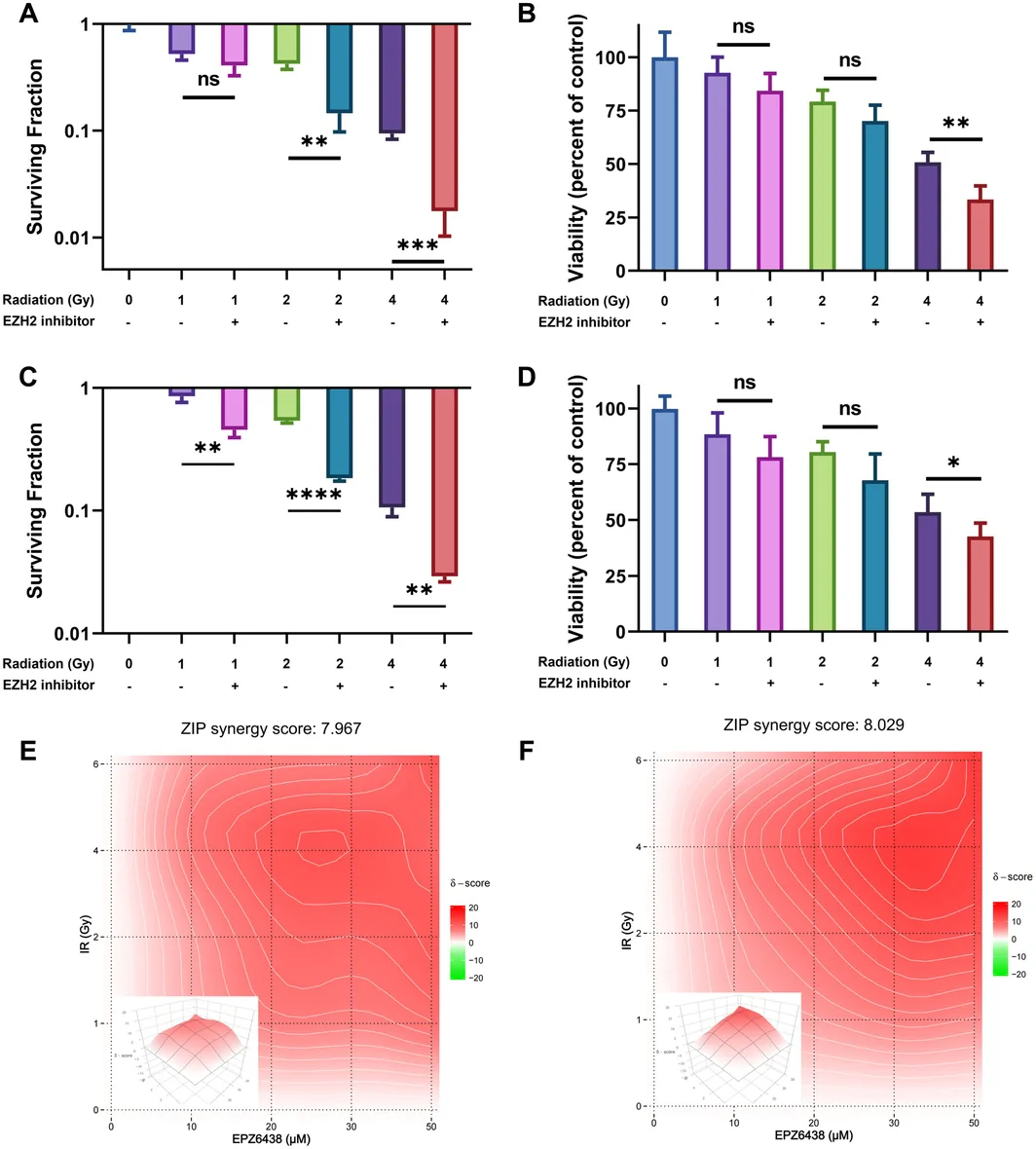
The effect of EPZ6438 on cell growth at different irradiation doses in (A, B) RM‐1 and (C, D) PC‐3. (A, C) Survival fraction; (B, D) Cell viability. Synergistic effect of EPZ6438 with irradiation in (E) RM‐1 and (F) PC‐3. The concentration of EPZ‐6438 in A–D is 30 μM. ns represents no significant difference; * represents *p* < 0.05; ** represents *p* < 0.01; *** represents *p* < 0.001; **** represents *p* < 0.0001.

Building on prior concentration optimisation (Section 3.1, 3.3), EPZ‐6438 was fixed at 30 μM for RM‐1 and PC‐3 cells to screen radiation doses (0, 1, 2, 4 Gy), quantifying SF and cell viability. In RM‐1, SF remained unchanged at 1 Gy (ns) but significantly decreased at 2 Gy (*p* < 0.05) and 4 Gy (*p* < 0.01) (Figure 2A), while viability showed no reduction at ≤ 2 Gy but significant suppression at 4 Gy (*p* < 0.01) (Figure 2B). For PC‐3, SF was significantly reduced at all doses (1, 2, 4 Gy, *p* < 0.05) (Figure 2C), whereas viability decreased only at 4 Gy (*p* < 0.01) (Figure 2D). Collectively, 4 Gy irradiation with 30 μM EPZ‐6438 demonstrated significant growth inhibition in both lines.

### 3.5 Synergistic Effect Validation

Although prior findings (Sections 3.1, 3.4) indicated 30 μM/4 Gy as a candidate optimal regimen, those unidimensional models (fixed drug/dose variables) failed to capture radiation‐drug synergy. Dose‐effect matrix analysis revealed peak synergy scores for RM‐1 at 25–30 μM with around 4 Gy irradiation (Figure 2E), and for PC‐3 at 30–40 μM with 3–5 Gy irradiation (Figure 2F). The 30 μM/4 Gy combination resided within both synergistic peaks, confirming its status as the optimal regimen subsequently adopted for all in vitro experiments.

### 3.6 In Vitro Migration and Invasion

**FIGURE 3:**
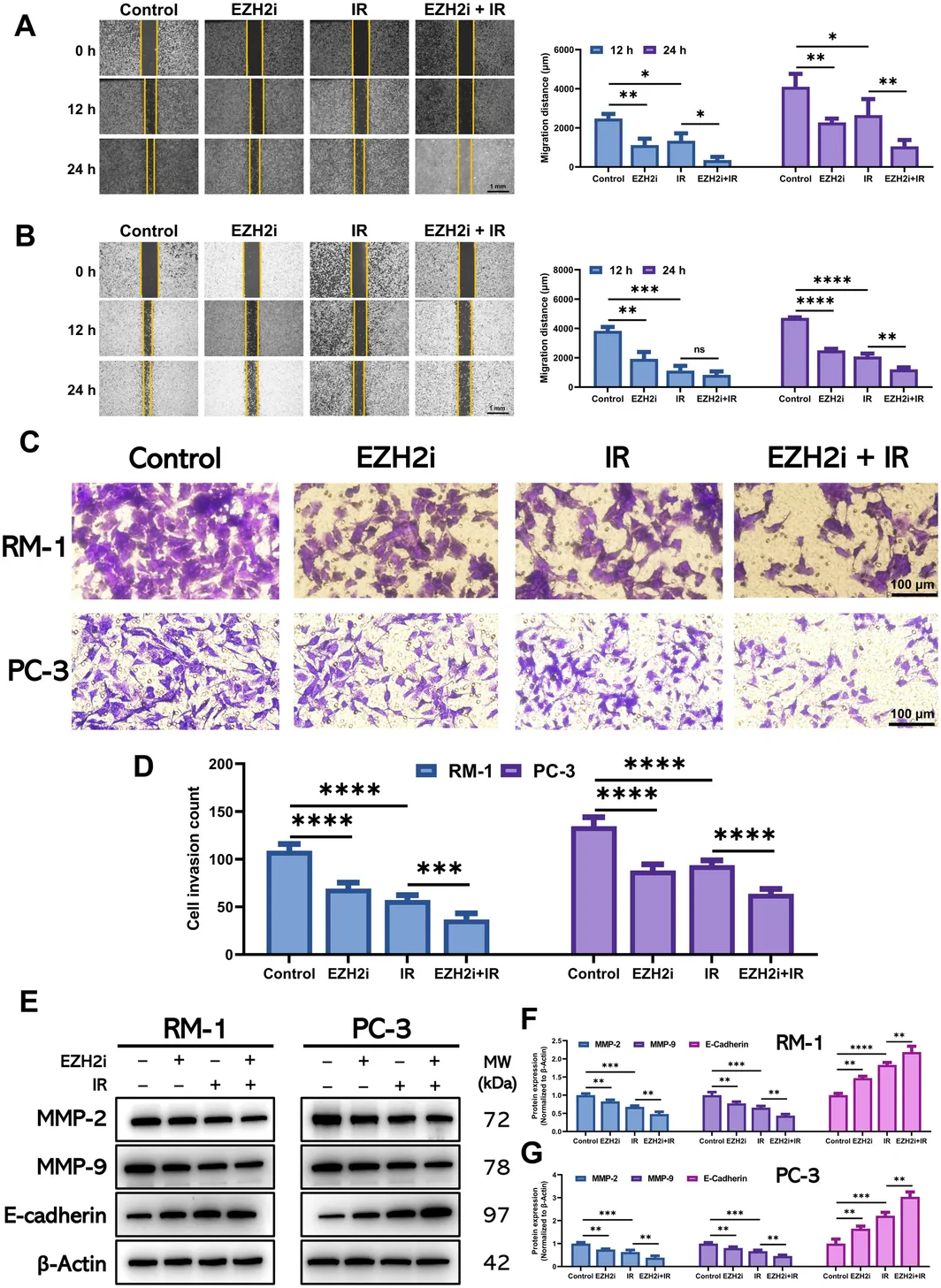
The effect of EZH2i combined with irradiation on the migration ability of (A) RM‐1 and (B) PC‐3 in vitro; Scale bar = 1 mm. The effect of EZH2i combined with irradiation on the invasion ability in vitro; (C, D) Transwell, (E–G) Western Blot; Scale bar = 100 μm. The concentration of EZH2i is 30 μM, and the IR dose is 4 Gy. ns represents no significant difference; * represents *p* < 0.05; ** represents *p* < 0.01; *** represents *p* < 0.001; **** represents *p* < 0.0001.

At 12/24 h post‐treatment, both EPZ‐6438 monotreatment and radiation alone significantly reduced migration in RM‐1 and PC‐3. Combination treatment enhanced suppression vs. radiation alone in RM‐1 at both time points (Figure 3A), while in PC‐3 this enhancement was only significant at 24 h (Figure 3B).

Transwell assays demonstrated significantly inhibited invasion by EPZ‐6438 or radiation monotreatment in both RM‐1 and PC‐3, with combination treatment providing further suppression vs. radiation alone (Figure 3C,D). WB confirmed decreased MMP‐2/−9 expression and increased E‐cadherin levels under monotreatments, effects significantly enhanced by combination treatment (Figure 3E–G), collectively validating the further anti‐invasive efficacy of the combined regimen.

### 3.7 In Vitro Proliferation and Apoptosis

**FIGURE 4:**
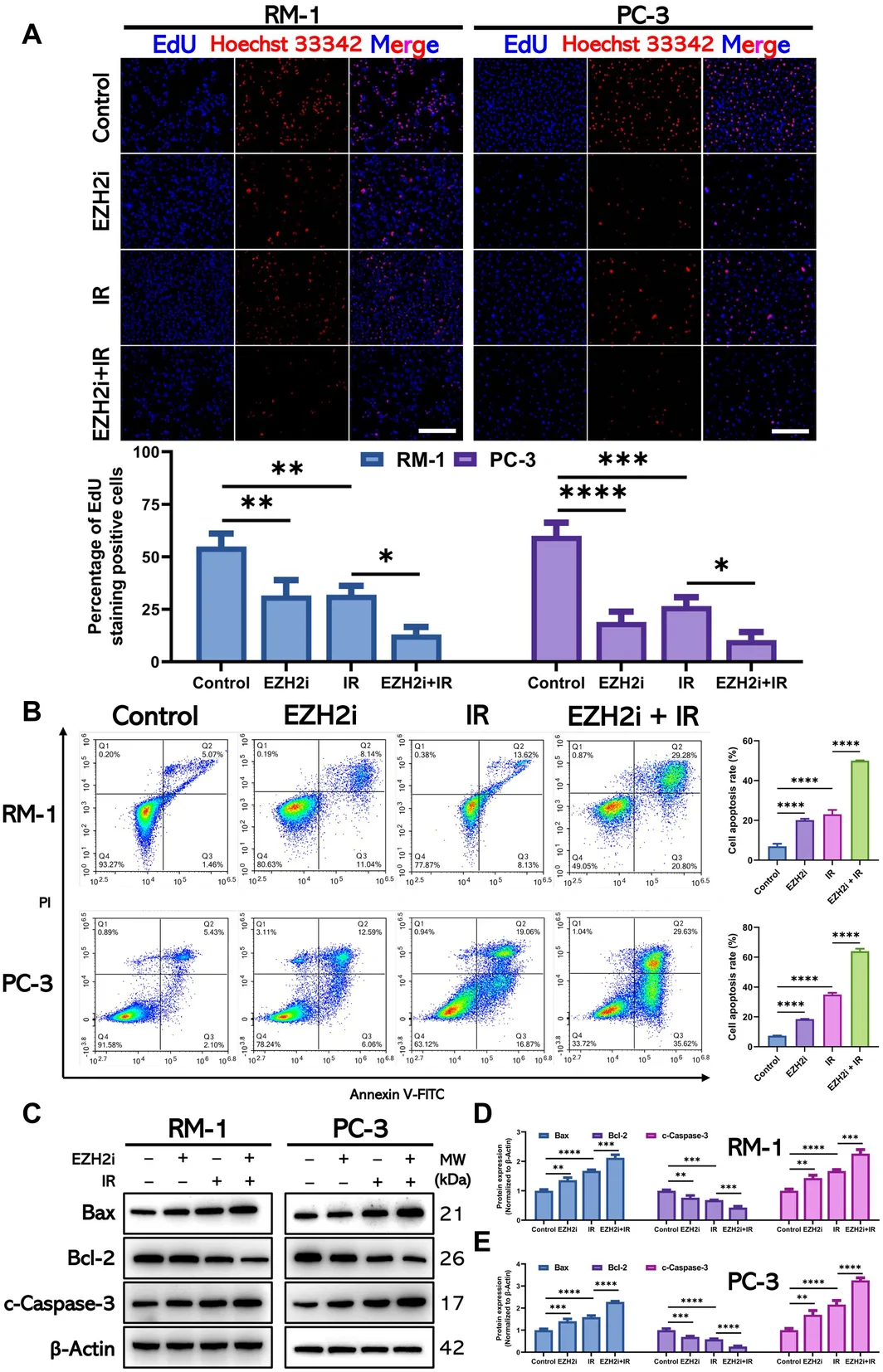
(A) The effect of EZH2i combined with irradiation on in vitro cell proliferation; Scale bar = 200 μm. The effect of EZH2i combined with irradiation on apoptosis in vitro; (B) Flow cytometry; (C–E) Western Blot. The concentration of EZH2i is 30 μM, and the IR dose is 4 Gy. * represents *p* < 0.05, ** represents *p* < 0.01, *** represents *p* < 0.001, **** represents *p* < 0.0001.

EdU assays demonstrated significantly reduced proliferation in both RM‐1 and PC‐3 cells following EPZ‐6438 or radiation monotreatment, with combination treatment further suppressing proliferation vs. radiation alone (Figure 4A).

Flow cytometry revealed significantly increased apoptosis under monotreatments, further enhanced by combination treatment (Figure 4B); WB confirmed these findings, showing monotreatment‐induced decreased Bcl‐2 expression, increased Bax and cleaved caspase‐3, effects further enhanced by combination treatment (Figure 4C–E), collectively validating the pro‐apoptotic synergy of the combined regimen.

### 3.8 DNA Damage

**FIGURE 5:**
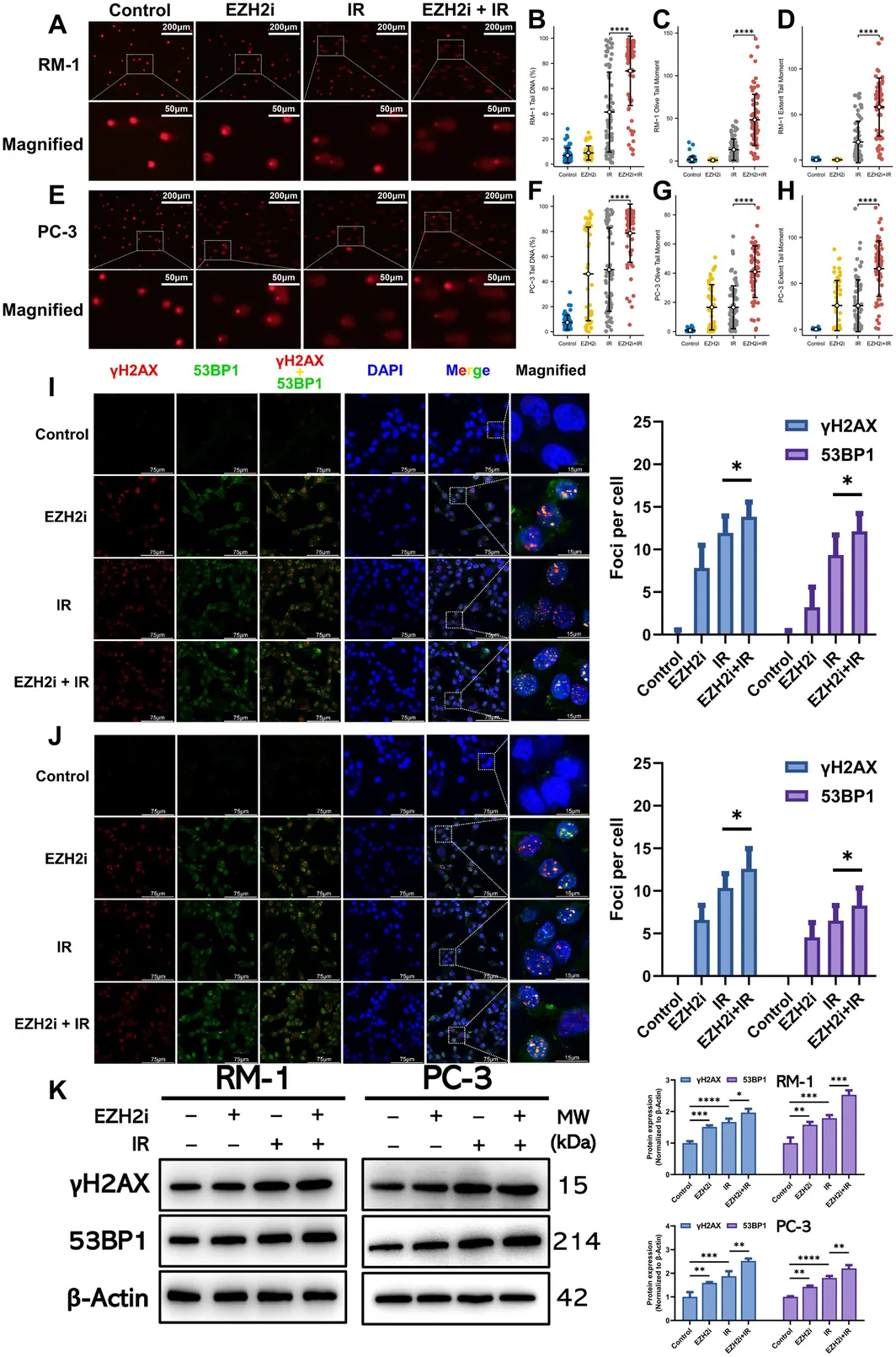
DNA damage to (A–D) RM‐1 and (E–H) PC‐3 in vitro by EZH2i combined with irradiation in comet assay; scale bar = 200 or 50 μm. DNA damage in (I) RM‐1 and (J) PC‐3 in vitro by EZH2i combined with irradiation in immunofluorescence and (K) Western Blot; scale bar = 75 or 15 μm. In panels B and F, 20%–40% indicates mild damage, 40%–95% indicates high damage, and > 95% indicates severe damage. The concentration of EZH2i is 30 μM, and the IR dose is 4 Gy. Statistical comparisons in panels B–D and F–J were performed using the two‐stage linear step‐up procedure of Benjamini, Krieger and Yekutieli. * represents *p* < 0.05, ** represents *p* < 0.01, *** represents *p* < 0.001, **** represents *p* < 0.0001.

Comet assays revealed significantly elevated tail DNA%, Olive tail moment, and extent tail moment in irradiated RM‐1 and PC‐3 cells, with combination further increasing these parameters and inducing severe DNA damage (tail DNA%, 40%–95%; Figure 5A–H). Immunofluorescence at 2 h post‐treatment demonstrated significantly increased γH2AX and 53BP1 foci formation with monotreatments, further enhanced by combination (Figure 5I,J). Concordantly, WB confirmed monotreatment‐induced upregulation of γH2AX and 53BP1 enhanced under combination treatment (Figure 5K).

### 3.9 Tumour Growth

**FIGURE 6:**
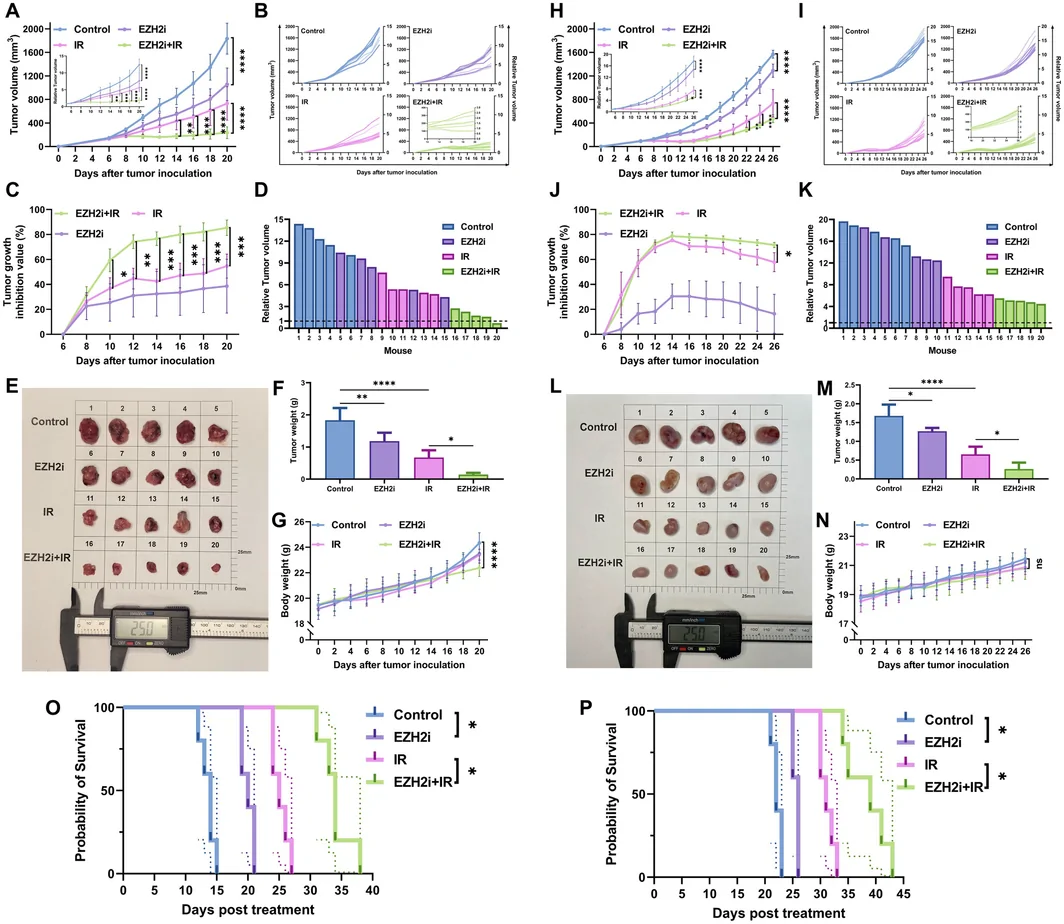
The effect of EZH2i combined with irradiation on tumour growth of (A–G) RM‐1 and (H–N) PC‐3 in vivo. (A, H) TV and RTV curves; (B, I) Individual TV and RTV curves (semi‐transparent for RTV, right Y‐axis); (C, J) Tumour growth inhibition curve; (D, K) Waterfall plot of individualised baseline response levels; (E, L) Ex vivo tumour tissue; (F, M) Tumour weight; (G, N) Body weight curve. Survival curves in (O) C57BL/6J/RM‐1 and (P) BALB/c‐nu/PC‐3 models. Localised tumour irradiation (4 Gy/fraction) was administered on days 0, 3, and 6; EZH2i (EPZ‐6438, 50 mg/kg) was delivered via daily intraperitoneal injection commencing on day 0. In panels A–N, ns represents no significant difference; * represents *p* < 0.05, ** represents *p* < 0.01, *** represents *p* < 0.001, **** represents *p* < 0.0001. In panels O and P, survival time was calculated from the first intervention; * represents log‐rank *p* < 0.05, with Bonferroni correction applied to all comparisons. PTV, tumour volume; RTV, relative tumour volume.

In C57BL/6J mice bearing RM‐1, combination treatment (50 mg/kg + 4 Gy) significantly reduced TV and RTV vs. monotreatment from day 14 (Figure 6A). Individual growth curves demonstrated stable tumour control in the combination group (Figure 6B), while TGI significantly increased from day 10 (Figure 6C). Waterfall analysis revealed a 93.3% (14/15) response rate at endpoint, with superior efficacy in the combination group (Figure 6D). Ex vivo tumours confirmed significant weight reduction in monotreatments, further decreased by combination (Figure 6E,F). Body weight remained consistent except for endpoint divergence in combination vs. control (Figure 6G).

In BALB/c‐nu/PC‐3 models, combination treatment reduced TV vs. radiation alone from day 22, while RTV from day 24 (Figure 6H), with stable growth curves (Figure 6I) and increased TGI at endpoint (Figure 6J). Waterfall plots showed an 86.7% (13/15) response rate with intervention efficacy (Figure 6K). Tumour weights decreased significantly under monotreatments, further reduced by combination (Figure 6L,M). Body weights remained comparable across groups (Figure 6N).

### 3.10 Survival Analysis

In independent cohorts, both EPZ‐6438 monotreatment and radiation alone significantly extended survival in C57BL/6J/RM‐1 (Figure 6O) and BALB/c‐nu/PC‐3 models (Figure 6P). The combined regimen conferred further survival advantage vs. monotreatment.

### 3.11 In Vivo Invasion

**FIGURE 7:**
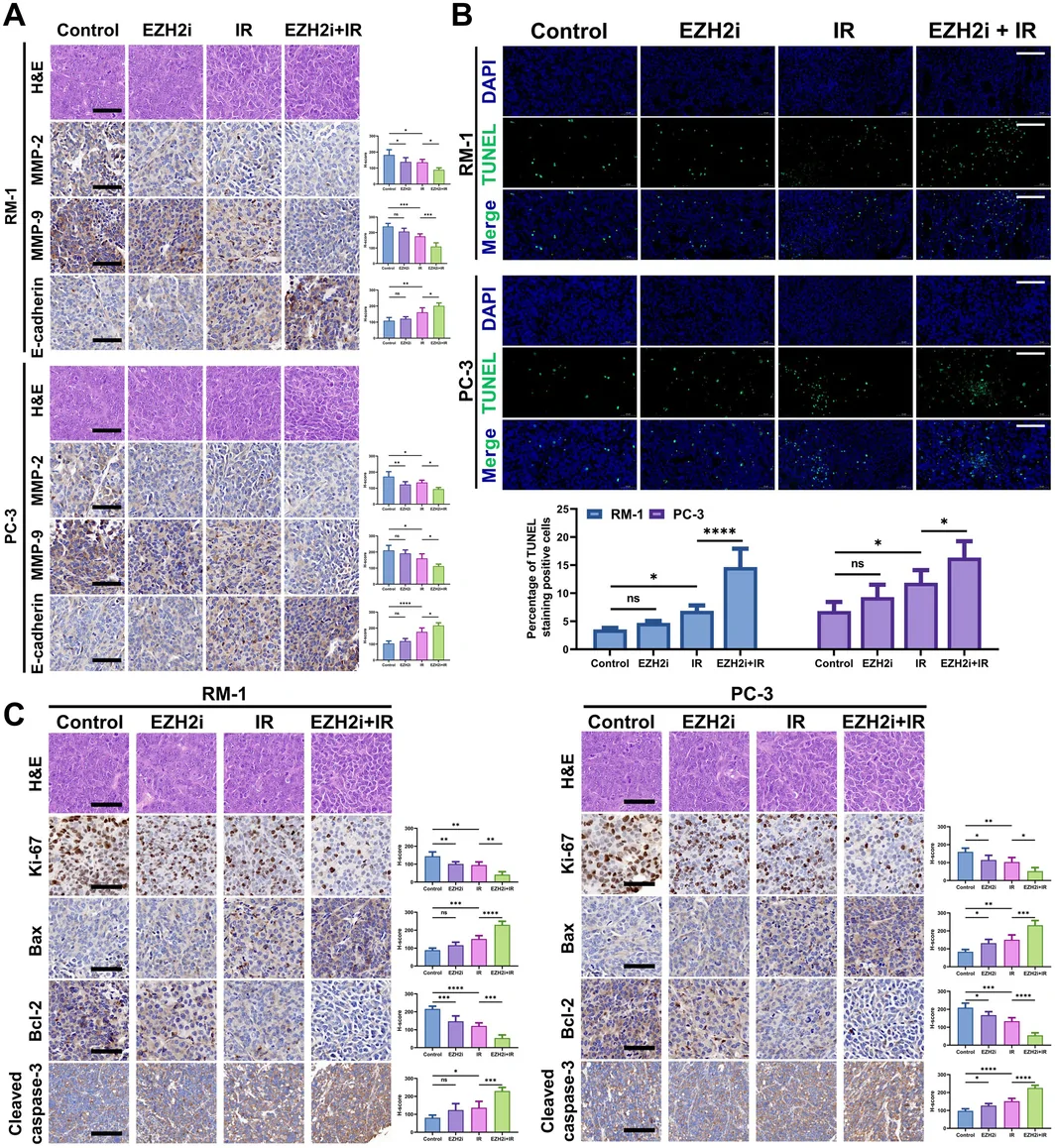
(A) The effect of EZH2i combined with irradiation on the expression of invasion‐related proteins in vivo; scale bar = 50 μm. (B) The effect of EZH2i combined with irradiation on apoptosis in vivo by TUNEL assay; scale bar = 100 μm. (C) The effect of EZH2i combined with irradiation on the expression of proliferation‐ and apoptosis‐related proteins in vivo; scale bar = 50 μm. The concentration of EZH2i is 30 μM, and the IR dose is 4 Gy. ns represents no significant difference; * represents *p* < 0.05, ** represents *p* < 0.01, *** represents *p* < 0.001, **** represents *p* < 0.0001.

Immunocytochemistry of tumour tissues revealed that in both models, EPZ‐6438 monotreatment significantly reduced MMP‐2 expression, while radiation alone decreased MMP‐2/−9 and increased E‐cadherin levels. The combined regimen further downregulated MMP‐2/−9 and upregulated E‐cadherin compared to radiation alone (Figure 7A).

### 3.12 In Vivo Apoptosis and Proliferation

TUNEL assays demonstrated enhanced apoptosis in irradiated tumour cells, with the combined regimen further increasing this effect (Figure 7B). Immunohistochemistry revealed radiation‐induced decreased Bcl‐2 expression, increased Bax and cleaved caspase‐3 levels, changes enhanced under combination treatment. Ki‐67 staining confirmed synergistic suppression of tumour proliferation by the combined regimen (Figure 7C).

### 3.13 Toxicological Analysis

Given the combined chemical/X‐ray intervention in mouse models, despite using an established EZH2i dose and fractionated radiation (Q2D), potential synergistic toxicity remained unclear. We assessed common toxicities (cardiac/hepatic/renal/haematopoietic) via histopathological and haematological tests.

H&E, Masson, and Sirius Red staining revealed no structural abnormalities in the heart, liver, or kidney of both models. Normal structural organisation, morphological integrity, and cellular alignment were observed in myocardial fibres, hepatic portal triads, renal glomeruli, and tubules, without evidence of collagen fibre hyperplasia/deposition or inflammatory cell infiltration in the interstitium (Figure S3).

C57BL/6J model exhibited no significant differences in WBC (Figure S4A), RBC (Figure S4C), and platelet counts (Figure S4D), all within the normal range; a slight neutrophil decrease occurred in combination compared to EZH2i but was still within the normal range (Figure S4B). Biochemistry showed normal ALT (Figure S4E), BUN (Figure S4G), Cr (Figure S4H), and LDH (Figure S4I); AST was elevated in EZH2i vs. control and combination vs. IR (within normal range, Figure S4F). Compared to the control group, CK were elevated in both the EZH2i and combination groups; nevertheless, all groups remained below the lower reference limit of the normal range (Figure S4J). BALB/c‐nu model displayed uniformly normal CBC and biochemistry (Figure S4K–T) across groups.

Collectively, no evidence indicated cardiac, hepatic, renal, or haematopoietic toxicity from the combined regimen.

## 4 Discussion

In clinical practice, the significance of radiosensitisation lies in two key aspects: When it is impossible to increase the radiation dose to the target volume due to various constraints (typically normal tissue toxicity), the concurrent use of specific drugs can enhance the efficacy of radiotherapy; alternatively, to achieve equivalent biological effects, it allows reduction of radiation doses, thereby minimising damage to surrounding normal tissues and potentially enabling opportunities for re‐irradiation. However, this phenomenon does not arise from simple additive cytotoxicity between chemical agents and radiation. For example, concurrent chemoradiotherapy, widely used clinically, fundamentally operates by inducing DNA DSBs against the background of chemotherapy‐induced DNA aberrations or replication stress, though specific mechanisms vary across different drug‐radiation combinations [17]. Antimetabolite drugs represented by gemcitabine and 5‐fluorouracil (5‐FU) are extensively employed in concurrent chemoradiotherapy for gastrointestinal cancers. These agents deplete nucleotides through their active metabolites (dFdCDP/dFdCTP for gemcitabine, FdUMP/FdUTP for 5‐FU) and redistribute cell cycles to S‐phase to achieve radiosensitisation [21]. Although S‐phase is typically a radioresistant stage in the normal cell cycle, under the influence of gemcitabine and 5‐FU, S‐phase cells develop severe and slowly repaired radiation‐induced DNA damage. Specifically, they cause radiation‐induced SSBs to collide with replication forks, generating single‐ended DSBs [22, 23]. Platinum‐based drugs are also widely combined with radiotherapy in clinical practice. Their cytotoxicity stems from forming DNA crosslinks that inhibit DNA replication and cause DNA breaks. The radiosensitising mechanism of platinum drugs differs from that of gemcitabine or 5‐FU, as it does not depend on cell cycle redistribution. Taking cisplatin as an example: cisplatin‐DNA adducts form complex and slowly repairable two‐ended DNA DSBs near radiation‐induced breaks, while partially impeding non‐homologous end joining (NHEJ)‐mediated DSB repair [24, 25]. Similarly, temozolomide, though forming distinct adducts, shares the same mechanism as platinum agents without altering cell cycle redistribution.

In this study, we first determined the IC_10_ and IC_50_ values to establish the concentration gradient for EZH2i. Through clonogenic assays, we quantified SER and DRR at each concentration level, thereby identifying provisional candidate concentrations of EZH2i. Concurrently, we confirmed significant functional inhibition of EZH2 in both RM‐1 and PC‐3 cell lines at these concentrations. Subsequently, we combined these candidate concentrations with varying radiation doses (0–6 Gy) and comprehensively analysed SF and cell viability metrics to determine provisional candidate radiation doses. Critically, at this stage, we could not yet conclude that EZH2i possessed intrinsic radiosensitising properties. To address this, we constructed dose‐effect matrices and demonstrated that the candidate concentration‐dose combinations resided within peak synergy score regions (δ‐score maximum), confirming robust radiation‐drug synergy. These findings provide compelling evidence that the observed biological effects on PCa cells are not merely additive consequences of EZH2i and radiation but rather substantiate EZH2i's capacity as a bona fide radiosensitiser.

Extensive prior research has demonstrated that downregulation of EZH2 expression effectively suppresses proliferation, migration, and invasive capabilities while promoting apoptosis in diverse malignancies including breast cancer, cervical cancer, colorectal cancer, lung adenocarcinoma, glioblastoma multiforme, gastric cancer, and renal cell carcinoma, thereby impeding tumour progression [19, 26, 27, 28, 29, 30, 31, 32, 33, 34, 35]. Within the specific context of PCa, some studies have established EZH2's integral involvement in multiple biological processes governing both in vitro and in vivo tumour progression, wherein suppression of its expression or functional inhibition demonstrably attenuates disease malignancy [36, 37, 38]. Synthesising this foundational evidence with our earlier discovery of EZH2i‐mediated radiosensitisation, we conducted systematic validations to determine whether EZH2i augments radiation‐induced suppression of prostate cancer progression. Initially, through wound healing assays, we confirmed that EZH2i combined with irradiation significantly enhanced the suppression of cellular migration compared with irradiation monotreatment. Subsequently, via Transwell invasion assays coupled with analysis of invasion‐associated proteins, we documented that the combined regimen further inhibited invasive capacity, evidenced by decreased expression of MMP‐2/−9 alongside increased expression of E‐cadherin. It is critical to contextualise that MMP‐2/−9 facilitate cancer progression, angiogenesis, and recruitment of macrophages or other myeloid cells to metastatic sites, while E‐cadherin functions as a pivotal inhibitor of epithelial‐mesenchymal transition (EMT) [39]. Additionally, using EdU proliferation assays, we showed enhanced growth inhibition under combination treatment. Finally, by flow cytometry and assessment of apoptosis‐related proteins, we verified significantly increased apoptosis, characterised by elevated expression of pro‐apoptotic Bax, reduced expression of anti‐apoptotic Bcl‐2, and heightened levels of cleaved caspase‐3, which noting that Bax and Bcl‐2 represent antagonistic regulators of apoptosis while c‐Caspase‐3 serves as the common downstream effector executioner in multiple apoptotic pathways, occupying a central position in programmed cell death [40]. Concomitantly, we performed in vivo validation in subcutaneous tumour models through measurement of tumour volumes, immunohistochemical staining, and TUNEL assays. These investigations consistently corroborated our in vitro findings, with the added observation that this radiosensitivity‐enhanced suppression of tumour progression translated into statistically significant survival benefits.

It is established that radiation‐induced cell death primarily stems from DNA damage, particularly DSBs, and that inhibiting DNA repair pathways can potentiate radiation efficacy. DSB repair predominantly occurs through two pathways: NHEJ and homologous recombination (HR) [41]. DSB formation triggers phosphorylation modifications of numerous proteins, initiating damage signalling and DNA repair cascades. A critical event is phosphorylation of γH2AX, which signals DSB presence and recruits repair proteins to form ionising radiation‐induced foci (IRIFs) [42]. Existing research indicates that EZH2 depletion impairs DSB repair capacity and enhances cellular radiosensitivity [43], partially through negatively regulating RAI2 to suppress tumour cell DNA repair [44]. Mechanistically, EZH2 modulates DNA damage response in p53‐wildtype cells primarily via transcriptional repression of FBXO32, which binds and promotes proteasome‐mediated degradation of p21 [45].

To investigate whether EZH2i influences DSB severity in PCa cells, we performed immunofluorescence staining to visualise γH2AX foci within nuclei. We observed that EZH2i combined with irradiation significantly increased γH2AX foci formation (vs. irradiation alone), indicating elevated DSB burden and consequent enhancement of cellular radiosensitivity. Furthermore, 53BP1, another critical component in mammalian DSB signalling and repair, is rapidly recruited to chromatin surrounding break sites upon DSB detection. 53BP1 acts as a key positive regulator of NHEJ‐mediated DSB repair, protecting broken ends from resection by DNA end‐processing machinery. Meanwhile, NHEJ, as an error‐prone repair mechanism, is not conducive to the maintenance of genomic stability [46]. Analogous to γH2AX, 53BP1 foci formation can be tracked via immunofluorescence to monitor damaged chromatin domains. In our study, the EZH2i‐radiation combination significantly increased 53BP1 foci accumulation, demonstrating that EZH2i achieves radiosensitisation in PCa both by exacerbating DNA damage degree and by modulating DSB repair pathway dynamics. These findings were further validated through WB analysis of γH2AX and 53BP1 expression.

In addition to its established role in DNA damage repair, emerging evidence has positioned EZH2 as a critical regulator of prostate cancer lineage plasticity. Venkadakrishnan et al. demonstrated that EZH2 exerts lineage‐specific functions in prostate adenocarcinoma and neuroendocrine prostate cancer (NEPC), modulating bivalent genes that lead to upregulation of NEPC‐associated transcriptional drivers such as ASCL1 [47]. Notably, EZH2 inhibition in NEPC models resulted in forward differentiation rather than lineage reversal, highlighting the context‐dependent nature of EZH2 function across prostate cancer subtypes. In a complementary study, Singh et al. revealed a functional crosstalk between EZH2 and DNA methylation in mediating NEPC lineage plasticity [48]. EZH2 deletion or pharmacological inhibition led to genome‐wide rewiring of DNA methylation, characterised by hypomethylation and upregulation of neuroendocrine lineage genes. Conversely, DNMT1 deletion resulted in significant redistribution of H3K27me3, particularly affecting bivalent promoters. These findings collectively indicate that the functional consequences of EZH2 inhibition are highly lineage‐dependent, and that perturbation of one epigenetic layer can trigger reciprocal changes in the other. This epigenetic crosstalk may represent a mechanism of resistance to EZH2 inhibitor therapy, as DNA hypermethylation can compensate for the loss of H3K27me3 to maintain transcriptional repression of key genes. Integrating these insights with our own findings, we propose that the radiosensitising effect of EZH2i in prostate cancer may be influenced by the underlying lineage state and the compensatory epigenetic responses triggered by EZH2 inhibition. Future studies investigating the combination of EZH2i with radiotherapy should consider patient stratification based on tumour subtype and epigenetic profiles, as well as the potential need for combinatorial targeting of compensatory DNA methylation pathways to maximise therapeutic benefit.

Our mechanistic findings establish that EZH2i potentiates radiation efficacy through exacerbating DNA damage and impairing DSB repair. However, the extent of this radiosensitising effect appears to be critically dependent on experimental conditions, as illustrated by comparison with a previous study. Wu et al. previously reported that EZH2i with UNC1999 only modestly radiosensitised PCa cells and did not increase γH2AX foci formation in PC‐3 cells [20]. Several experimental factors may account for the apparent discrepancy between our findings and theirs. First, we employed a higher concentration of the selective EZH2i EPZ‐6438 (30 μM) compared to the 4.0 μM UNC1999 used in their PC‐3 experiments. Second, our radiation dose of 4 Gy, determined through systematic dose optimisation, is higher than the 2 Gy used in their study. Third, our continuous drug exposure protocol, as opposed to transient exposure (withdrawal 24 h post‐irradiation), may more effectively sustain the interference with DSB repair processes. Fourth, the distinct genetic backgrounds of PCa cell lines, particularly p53 status, may modulate the threshold at which EZH2i manifests as enhanced radiosensitivity. Collectively, these differences suggest that the radiosensitising effect of EZH2i is dose‐ and schedule‐dependent, and that our optimised regimen achieves more robust enhancement of radiation response. Importantly, while Wu et al. concluded that EZH2 function may play at most a modest role in promoting radioresistance, our in vitro and in vivo data demonstrate that under optimised conditions, EZH2i can produce substantial radiosensitisation, supporting the clinical translation of this strategy. We observed a noticeable difference in the magnitude of tumour volume reduction between the two in vivo models, with RM‐1 tumours showing a more pronounced response to the combination therapy compared to PC‐3 xenografts. We believe this disparity warrants discussion and may offer valuable insights for clinical translation. Several factors may contribute to this differential responsiveness. First, the immune microenvironment differs substantially between the two models: RM‐1 cells were inoculated into immunocompetent C57BL/6J mice, whereas PC‐3 cells were grown in T‐cell‐deficient BALB/c‐nu nude mice. Given that EZH2 inhibitors have been reported to modulate the tumour immune microenvironment—including CD8+ T cell infiltration, NK cell activity, and macrophage polarisation—the combination of EZH2i and radiotherapy in an immunocompetent setting may exert antitumour effects through dual mechanisms: Direct radiosensitisation of tumour cells and concurrent remodelling of the immune microenvironment [49]. In the absence of adaptive immunity in nude mice, the latter mechanism would be largely inoperative, which may partially explain the more modest effect observed in the PC‐3 model. Second, intrinsic biological differences between the cell lines, including EZH2 expression levels, dependence on EZH2 function, p53 status (RM‐1 is p53‐mutant while PC‐3 is p53‐null), and baseline DNA repair capacity, may also modulate the extent of radiosensitisation. This is consistent with our in vitro data, which already demonstrated a comparatively weaker radiosensitising effect in PC‐3 than in RM‐1 cells (Figure 2A–D). Third, differences in tumour growth kinetics—RM‐1 tumours grow more rapidly and thus provide a wider therapeutic window for intervention—may further amplify the absolute differences observed within the same treatment duration. Importantly, despite the more modest tumour volume reduction in the PC‐3 model, we consistently observed statistically significant and biologically meaningful benefits across multiple endpoints: Relative tumour volume (RTV) became significantly lower than that of the irradiation‐alone group from day 24 onward, tumour growth inhibition (TGI) was significantly elevated at endpoint, tumour weight was significantly reduced, and most critically, survival was significantly prolonged in the combination group (Figure 6P). These data demonstrate that even in an immunodeficient background, EZH2i can still confer clinically relevant therapeutic gains through direct radiosensitisation, albeit to a lesser extent than in immunocompetent settings. From a clinical translation perspective, these findings carry several implications: Patient stratification based on biomarkers such as EZH2 expression level, tumour immune infiltration status, and p53 mutation status may help identify those most likely to benefit; the addition of immune checkpoint inhibitors to the EZH2i‐radiotherapy regimen warrants exploration in immunocompetent settings; and survival endpoints may serve as more reliable indicators of therapeutic efficacy than tumour volume changes alone. Collectively, rather than representing a weakness of our study, the differential responses between the two models provide valuable insights into the mechanisms and optimal clinical application of EZH2i‐based radiosensitisation.

## 5 Conclusions

This study demonstrates that the EZH2i Tazemetostat (EPZ‐6438) significantly enhances the radiosensitivity of prostate cancer in vitro and in vivo. The combined regimen synergistically suppresses tumour proliferation, migration, and invasion while promoting apoptosis. Mechanistically, EPZ‐6438 potentiates radiotherapy efficacy by exacerbating DNA damage and affecting DSB repair pathways. These effects translate to suppressed tumour progression and extended survival in preclinical models, with no observed systemic toxicity. Our findings establish EZH2 inhibition as a promising therapeutic strategy to improve radiotherapeutic outcomes in PCa.

## Author Contributions

**Tian‐Qi Du:** conceptualization, data curation, formal analysis, investigation, methodology, resources, software, validation, visualization, writing – original draft, writing – review and editing. **Gang Chen:** conceptualization, funding acquisition, project administration, supervision, writing – review and editing. **Rui‐Ji Liu:** data curation, methodology, writing – original draft. **Yuhong Ou:** data curation, formal analysis, resources, validation, writing – original draft. **Yi Feng:** software, formal analysis, visualization, writing – original draft. **Yaxun Li:** formal analysis, writing – original draft. **Xingyu Wu:** conceptualization, supervision, writing – review and editing.

## Funding

This study was supported by the Science and Technology Plan Project of Yangjiang (No. 2024013).

## Ethics Statement

The animal study protocol was approved by the Animal Research and Ethics Committee of the Biomedical Center, Institute of Modern Physics, Chinese Academy of Sciences (No. 2023 (023)).

## Conflicts of Interest

The authors declare no conflicts of interest.

## Supporting information


**Table S1:** Key reagents and resources.
**Figure S1:** (A) Animal model intervention; (B) Flowchart.
**Figure S2:** EPZ‐6438‐mediated suppression of EZH2 activity.
**Figure S3:** H&E, Masson, and Sirius Red staining of heart, liver, and kidney in (A) C57BL/6J and (B) BALB/c‐nu. Scale bar = 200 μm.
**Figure S4:** Peripheral blood tests in (A–J) C57BL/6J and (K–T) BALB/c‐nu. (A, K) White blood cell, (B, L) Neutrophil, (C, M) Red blood cell, (D, N) Platelet, (E, O) Alanine aminotransferase, (F, P) Aspartate aminotransferase, (G, Q) Blood urea nitrogen, (H, R) Creatinine, (I, S) Lactate dehydrogenase, (J, T) Creatine kinase. The semi‐transparent cyan background represents the normal reference ranges for corresponding indicators of each mouse strain.; ns represents no significant difference, * represents p < 0.05, ** represents p < 0.01.

## Data Availability

The data that support the findings of this study are available from the corresponding author upon reasonable request.

## References

[1] K. Nepali and J. P. Liou , “Recent Developments in Epigenetic Cancer Therapeutics: Clinical Advancement and Emerging Trends,” Journal of Biomedical Science 28 (2021): 27. 33840388 10.1186/s12929-021-00721-x PMC8040241

[2] D. Pasini and L. Di Croce , “Emerging Roles for Polycomb Proteins in Cancer,” Current Opinion in Genetics & Development 36 (2016): 50–58. 27151431 10.1016/j.gde.2016.03.013

[3] A. He , X. Shen , Q. Ma , et al., “PRC2 Directly Methylates GATA4 and Represses Its Transcriptional Activity,” Genes & Development 26 (2012): 37–42. 22215809 10.1101/gad.173930.111 PMC3258964

[4] E. Kim , M. Kim , D. H. Woo , et al., “Phosphorylation of EZH2 Activates STAT3 Signaling via STAT3 Methylation and Promotes Tumorigenicity of Glioblastoma Stem‐Like Cells,” Cancer Cell 23 (2013): 839–852. 23684459 10.1016/j.ccr.2013.04.008 PMC4109796

[5] K. Xu , Z. J. Wu , A. C. Groner , et al., “EZH2 Oncogenic Activity in Castration‐Resistant Prostate Cancer Cells Is Polycomb‐Independent,” Science 338 (2012): 1465–1469. 23239736 10.1126/science.1227604 PMC3625962

[6] J. Kim , Y. Lee , X. Lu , et al., “Polycomb‐ and Methylation‐Independent Roles of EZH2 as a Transcription Activator,” Cell Reports 25 (2018): 2808–2820.e4. 30517868 10.1016/j.celrep.2018.11.035 PMC6342284

[7] J. Wang , X. Yu , W. Gong , et al., “EZH2 Noncanonically Binds cMyc and p300 Through a Cryptic Transactivation Domain to Mediate Gene Activation and Promote Oncogenesis,” Nature Cell Biology 24 (2022): 384–399. 35210568 10.1038/s41556-022-00850-x PMC9710513

[8] S. L. Nutt , C. Keenan , M. Chopin , and R. S. Allan , “EZH2 function in immune cell development,” Biological Chemistry 401 (2020): 933–943. 32045348 10.1515/hsz-2019-0436

[9] T. Ito , Y. V. Teo , S. A. Evans , N. Neretti , and J. M. Sedivy , “Regulation of Cellular Senescence by Polycomb Chromatin Modifiers Through Distinct DNA Damage‐ and Histone Methylation‐Dependent Pathways,” Cell Reports 22 (2018): 3480–3492. 29590617 10.1016/j.celrep.2018.03.002 PMC5915310

[10] J. Zeng , J. Zhang , Y. Sun , et al., “Targeting EZH2 for Cancer Therapy: From Current Progress to Novel Strategies,” European Journal of Medicinal Chemistry 238 (2022): 114419. 35569264 10.1016/j.ejmech.2022.114419

[11] R. Duan , W. Du , and W. Guo , “EZH2: A Novel Target for Cancer Treatment,” Journal of Hematology & Oncology 13 (2020): 104. 32723346 10.1186/s13045-020-00937-8 PMC7385862

[12] Y. Yi , Y. Li , C. Li , et al., “Methylation‐Dependent and ‐Independent Roles of EZH2 Synergize in CDCA8 Activation in Prostate Cancer,” Oncogene 41 (2022): 1610–1621. 35094010 10.1038/s41388-022-02208-x PMC9097394

[13] Y. Yi , Y. Li , Q. Meng , et al., “A PRC2‐Independent Function for EZH2 in Regulating rRNA 2'‐O Methylation and IRES‐Dependent Translation,” Nature Cell Biology 23 (2021): 341–354. 33795875 10.1038/s41556-021-00653-6 PMC8162121

[14] A. E. Koyen , M. Z. Madden , D. Park , et al., “EZH2 Has a Non‐Catalytic and PRC2‐Independent Role in Stabilizing DDB2 to Promote Nucleotide Excision Repair,” Oncogene 39 (2020): 4798–4813. 32457468 10.1038/s41388-020-1332-2 PMC7305988

[15] W. Burkart , T. Jung , and G. Frasch , “Damage Pattern as a Function of Radiation Quality and Other Factors,” Comptes Rendus de l'Academie des Sciences. Serie III, Sciences de la Vie 322 (1999): 89–101. 10.1016/s0764-4469(99)80029-8 10196658

[16] E. Mladenov , S. Magin , A. Soni , and G. Iliakis , “DNA Double‐Strand Break Repair as Determinant of Cellular Radiosensitivity to Killing and Target in Radiation Therapy,” Frontiers in Oncology 3 (2013): 113. 23675572 10.3389/fonc.2013.00113 PMC3650303

[17] M. A. Morgan , L. A. Parsels , J. Maybaum , and T. S. Lawrence , “Improving the Efficacy of Chemoradiation With Targeted Agents,” Cancer Discovery 4 (2014): 280–291. 24550033 10.1158/2159-8290.CD-13-0337 PMC3947675

[18] X. Zhang , X. Ma , Q. Wang , and Z. Kong , “EZH2 Targeting to Improve the Sensitivity of Acquired Radio‐Resistance Bladder Cancer Cells,” Translational Oncology 16 (2022): 101316. 34952334 10.1016/j.tranon.2021.101316 PMC8695351

[19] F. Han , D. Huang , J. Meng , J. Chu , M. Wang , and S. Chen , “miR‐126‐5p Enhances Radiosensitivity of Lung Adenocarcinoma Cells by Inhibiting EZH2 via the KLF2/BIRC Axis,” Journal of Cellular and Molecular Medicine 26 (2022): 2529–2542. 35322532 10.1111/jcmm.17135 PMC9077299

[20] X. Wu , H. Scott , S. V. Carlsson , et al., “Increased EZH2 Expression in Prostate Cancer Is Associated With Metastatic Recurrence Following External Beam Radiotherapy,” Prostate 79 (2019): 1079–1089. 31104332 10.1002/pros.23817 PMC6563086

[21] C. J. McGinn , D. S. Shewach , and T. S. Lawrence , “Radiosensitizing nucleosides,” Journal of the National Cancer Institute 88 (1996): 1193–1203. 8780628 10.1093/jnci/88.17.1193

[22] P. Groth , M. L. Orta , I. Elvers , M. M. Majumder , A. Lagerqvist , and T. Helleday , “Homologous Recombination Repairs Secondary Replication Induced DNA Double‐Strand Breaks After Ionizing Radiation,” Nucleic Acids Research 40 (2012): 6585–6594. 22505579 10.1093/nar/gks315 PMC3413124

[23] D. Branzei and M. Foiani , “Regulation of DNA Repair Throughout the Cell Cycle,” Nature Reviews. Molecular Cell Biology 9 (2008): 297–308. 18285803 10.1038/nrm2351

[24] C. R. Sears and J. J. Turchi , “Complex Cisplatin‐Double Strand Break (DSB) Lesions Directly Impair Cellular Non‐Homologous End‐Joining (NHEJ) Independent of Downstream Damage Response (DDR) Pathways,” Journal of Biological Chemistry 287 (2012): 24263–24272. 22621925 10.1074/jbc.M112.344911 PMC3397852

[25] G. D. Wilson , S. M. Bentzen , and P. M. Harari , “Biologic Basis for Combining Drugs With Radiation,” Seminars in Radiation Oncology 16 (2006): 2–9. 16378901 10.1016/j.semradonc.2005.08.001

[26] L. Zhang , J. Qu , Y. Qi , et al., “EZH2 Engages TGFβ Signaling to Promote Breast Cancer Bone Metastasis via Integrin β1‐FAK Activation,” Nature Communications 13 (2022): 2543. 10.1038/s41467-022-30105-0 PMC9091212 35538070

[27] R. Chen , Q. Gan , S. Zhao , et al., “DNA Methylation of miR‐138 Regulates Cell Proliferation and EMT in Cervical Cancer by Targeting EZH2,” BMC Cancer 22 (2022): 488. 35505294 10.1186/s12885-022-09477-5 PMC9063191

[28] J. Yu , K. Yang , J. Zheng , et al., “Activation of FXR and Inhibition of EZH2 Synergistically Inhibit Colorectal Cancer Through Cooperatively Accelerating FXR Nuclear Location and Upregulating CDX2 Expression,” Cell Death & Disease 13 (2022): 388. 35449124 10.1038/s41419-022-04745-5 PMC9023572

[29] C. Li , J. Song , Z. Guo , et al., “EZH2 Inhibitors Suppress Colorectal Cancer by Regulating Macrophage Polarization in the Tumor Microenvironment,” Frontiers in Immunology 13 (2022): 857808. 35432300 10.3389/fimmu.2022.857808 PMC9010515

[30] L. C. Liu , Y. C. Chien , G. W. Wu , et al., “Analysis of EZH2 Genetic Variants on Triple‐Negative Breast Cancer Susceptibility and Pathology,” International Journal of Medical Sciences 19 (2022): 1023–1028. 35813302 10.7150/ijms.71931 PMC9254368

[31] A. E. Yetişir , S. Paydaş , M. Büyükşimşek , et al., “Effects of Enhancer of Zeste Homolog 2 and Mucin 1 Expressions on Treatment Response in Breast Cancer,” Revista da Associacao Medica Brasileira (1992) 69 (2023): 153–158. 36820722 10.1590/1806-9282.20221123 PMC9937598

[32] M. Paskeh , A. Mehrabi , M. H. Gholami , et al., “EZH2 as a New Therapeutic Target in Brain Tumors: Molecular Landscape, Therapeutic Targeting and Future Prospects,” Biomedicine & pharmacotherapy = Biomedecine & pharmacotherapie 146 (2022): 112532. 34906772 10.1016/j.biopha.2021.112532

[33] A. Del Moral‐Morales , J. C. González‐Orozco , A. M. Hernández‐Vega , K. Hernández‐Ortega , K. M. Peña‐Gutiérrez , and I. Camacho‐Arroyo , “EZH2 Mediates Proliferation, Migration, and Invasion Promoted by Estradiol in Human Glioblastoma Cells,” Front Endocrinol (Lausanne) 13 (2022): 703733. 35197928 10.3389/fendo.2022.703733 PMC8859835

[34] J. Zhao , W. Jin , K. Yi , et al., “Combination LSD1 and HOTAIR‐EZH2 Inhibition Disrupts Cell Cycle Processes and Induces Apoptosis in Glioblastoma Cells,” Pharmacological Research 171 (2021): 105764. 34246782 10.1016/j.phrs.2021.105764

[35] Z. Chen , L. Yuan , X. Li , J. Yu , and Z. Xu , “BMP2 Inhibits Cell Proliferation by Downregulating EZH2 in Gastric Cancer,” Cell Cycle 21 (2022): 2298–2308. 35856444 10.1080/15384101.2022.2092819 PMC9586658

[36] L. Guo , X. Luo , P. Yang , et al., “Ilicicolin A Exerts Antitumor Effect in Castration‐Resistant Prostate Cancer via Suppressing EZH2 Signaling Pathway,” Frontiers in Pharmacology 12 (2021): 723729. 34776951 10.3389/fphar.2021.723729 PMC8578973

[37] Z. Jing , Q. Liu , W. Xie , et al., “NCAPD3 Promotes Prostate Cancer Progression by Up‐Regulating EZH2 and MALAT1 Through STAT3 and E2F1,” Cellular Signalling 92 (2022): 110265. 35085770 10.1016/j.cellsig.2022.110265

[38] S. H. Park , K. W. Fong , J. Kim , et al., “Posttranslational Regulation of FOXA1 by Polycomb and BUB3/USP7 Deubiquitin Complex in Prostate Cancer,” Science Advances 7 (2021): eabe2261. 33827814 10.1126/sciadv.abe2261 PMC8026124

[39] B. Psaila and D. Lyden , “The Metastatic Niche: Adapting the Foreign Soil,” Nature Reviews. Cancer 9 (2009): 285–293. 19308068 10.1038/nrc2621 PMC3682494

[40] S. Pang , J. D'Rozario , S. Mendonca , et al., “Mesenchymal Stromal Cell Apoptosis Is Required for Their Therapeutic Function,” Nature Communications 12 (2021): 6495. 10.1038/s41467-021-26834-3 PMC8586224 34764248

[41] C. Wyman and R. Kanaar , “DNA Double‐Strand Break Repair: All's Well That Ends Well,” Annual Review of Genetics 40 (2006): 363–383. 10.1146/annurev.genet.40.110405.090451 16895466

[42] I. Y. Belyaev , “Radiation‐Induced DNA Repair Foci: Spatio‐Temporal Aspects of Formation, Application for Assessment of Radiosensitivity and Biological Dosimetry,” Mutation Research 704 (2010): 132–141. 20096808 10.1016/j.mrrev.2010.01.011

[43] S. Campbell , I. H. Ismail , L. C. Young , G. G. Poirier , and M. J. Hendzel , “Polycomb Repressive Complex 2 Contributes to DNA Double‐Strand Break Repair,” Cell Cycle 12 (2013): 2675–2683. 23907130 10.4161/cc.25795 PMC3865057

[44] M. Huang , J. Ding , X. Wu , et al., “EZH2 Affects Malignant Progression and DNA Damage Repair of Lung Adenocarcinoma Cells by Regulating RAI2 Expression,” Mutation Research 825 (2022): 111792. 35939884 10.1016/j.mrfmmm.2022.111792

[45] Z. Wu , S. T. Lee , Y. Qiao , et al., “Polycomb Protein EZH2 Regulates Cancer Cell Fate Decision in Response to DNA Damage,” Cell Death and Differentiation 18 (2011): 1771–1779. 21546904 10.1038/cdd.2011.48 PMC3190113

[46] S. Panier and S. J. Boulton , “Double‐Strand Break Repair: 53BP1 Comes Into Focus,” Nature Reviews. Molecular Cell Biology 15 (2014): 7–18. 24326623 10.1038/nrm3719

[47] V. B. Venkadakrishnan , A. G. Presser , R. Singh , et al., “Lineage‐Specific Canonical and Non‐Canonical Activity of EZH2 in Advanced Prostate Cancer Subtypes,” Nature Communications 15 (2024): 6779. 10.1038/s41467-024-51156-5 PMC11310309 39117665

[48] R. Singh , V. B. Venkadakrishnan , E. Imada , et al., “Crosstalk Between EZH2 and DNA Methylation Mediates Neuroendocrine Prostate Cancer Lineage Plasticity,” Nature Communications 17 (2026): 17. 10.1038/s41467-026-69308-0 PMC13036044 41720768

[49] T. Q. Du , R. Liu , Q. Zhang , et al., “EZH2 as a Prognostic Factor and Its Immune Implication With Molecular Characterization in Prostate Cancer: An Integrated Multi‐Omics in Silico Analysis,” Biomolecules 12 (2022): 1617. 36358967 10.3390/biom12111617 PMC9687944

