# Supplementary material for: Unveiling the New Trick for Epigenetic Drug: Radiation Enhancement of Prostate Cancer by the EZH2 Inhibitor Tazemetostat
Source: J Cell Mol Med. 2026 Aug 19;30(16):e71330. doi: 10.1111/jcmm.71330 (PMC13490720; doi:10.1111/jcmm.71330)
Supplement: Supplementary file 1 [file JCMM-30-e71330-s001.docx]

Supplementary Materials

**Table S1.** Key reagent and resource

| **Key Reagent and Resource** | **Manufacturer or Origin** | **Cat#** | **RRID** |
| --- | --- | --- | --- |
| **Antibodies** |  |  |  |
| Beta Actin Monoclonal antibody | Proteintech | 66009-1-lg | AB_2782959 |
| EZH2 Monoclonal antibody | Proteintech | 66476-1-lg | AB_2881842 |
| Histone H3 Polyclonal antibody | Proteintech | 17168-1-AP | AB_2716755 |
| Histone H3K27me3 (Tri-methyl Lys27) antibody | GeneTex | GTX129774 | AB_2886084 |
| Histone H2A.X antibody | GeneTex | GTX108272 | AB_1950472 |
| 53BP1 antibody [HL275] | GeneTex | GTX635479 | AB_2888536 |
| MMP2 Polyclonal antibody | Proteintech | 10373-2-AP | AB_2250823 |
| MMP9 (N-terminal) Polyclonal antibody | Proteintech | 10375-2-AP | AB_10897178 |
| E-cadherin Polyclonal antibody | Proteintech | 20874-1-AP | AB_10697811 |
| BCL2 Monoclonal antibody | Proteintech | 68103-1-lg | AB_2923635 |
| human BCL2 Polyclonal antibody | Proteintech | 12789-1-AP | AB_2227948 |
| BAX Polyclonal antibody | Proteintech | 50599-2-lg | AB_2061561 |
| Caspase 3 (cleaved Asp175) antibody | GeneTex | GTX86952 | AB_10722506 |
| ki67 Polyclonal antibody | Proteintech | 28074-1-AP | AB_2918145 |
| KI67 Polyclonal antibody | Proteintech | 27309-1-AP | AB_2756525 |
| HRP-conjugated Affinipure Goat Anti-Rabbit IgG(H+L) | Proteintech | SA00001-2 | AB_2722564 |
| HRP-conjugated Affinipure Goat Anti-Mouse IgG(H+L) | Proteintech | SA00001-1 | AB_2722565 |
| **EZH2 inhibatior** |  |  |  |
| EPZ-6438 (Tazemetostat) | GlpBio | GC14062 | ‒ |
| **Cell lines** |  |  |  |
| RM-1 (*Mus musculus,* Mouse) | ATCC | CRL- 3310 | CVCL_B459 |
| PC-3 (*Homo sapiens*, Human) | ATCC | CVCL_0035 | CVCL_0035 |
| **Mice models** |  |  |  |
| C57BL/6J | Lanzhou Veterinary Research Institute, Chinese Academy of Agricultural Sciences | ‒ | MGI:3028467 |
| BALB/c-nu (Crl:NU(NCr)-Foxn1^nu^) | Lanzhou Veterinary Research Institute, Chinese Academy of Agricultural Sciences | ‒ | IMSR_CRL:490 |
| **Software and algorithms** |  | **URL** |  |
| R v3.6.3 (R Project for Statistical Computing) | R Development Core Team | http://www.r-project.org/ | SCR_001905 |
| GraphPad Prism v9.3.0 | GraphPad | http://www.graphpad.com/ | SCR_002798 |
| SynergyFinder web application | A. Ianevski et al. [1] | https://synergyfinder.fimm.fi | SCR_026127 |
| Fiji v2.9.0 | Fiji team | http://fiji.sc | SCR_002285 |

**Reference**

1.	Ianevski, A.; Giri, A.K.; Aittokallio, T. SynergyFinder 3.0: an interactive analysis and consensus interpretation of multi-drug synergies across multiple samples. *Nucleic Acids Res.* **2022**, *50,* W739-W743.

**Figure S1.** (**A**) Animal model intervention; (**B**) Flowchart.

**Figure S2.** EPZ-6438 mediated suppression of EZH2 activity.

**Figure S3.** H&E, Masson, and Sirius Red staining of heart, liver, and kidney in (**A**) C57BL/6J and (**B**) BALB/c-nu. Scale bar = 200 μm.

**Figure S4.** Peripheral blood tests in (**A-J**) C57BL/6J and (**K-T**) BALB/c-nu. (**A,K**) White blood cell, (**B,L**) Neutrophil, (**C,M**) Red blood cell, (**D,N**) Platelet, (**E,O**) Alanine aminotransferase, (**F,P**) Aspartate aminotransferase, (**G,Q**) Blood urea nitrogen, (**H,R**) Creatinine, (**I,S**) Lactate dehydrogenase, (**J,T**) Creatine kinase. The semi-transparent cyan background represents the normal reference ranges for corresponding indicators of each mouse strain.; ns represents no significant, * represents *p* < 0.05, ** represents *p* < 0.01.
